# The future disposition Inventory-24: reliability and validity estimates in a large sample of Chinese University students

**DOI:** 10.1186/s12888-018-1875-8

**Published:** 2018-09-17

**Authors:** Lu Yuan, Dong-Fang Wang, Bob Lew, Augustine Osman, Cun-Xian Jia

**Affiliations:** 1 Department of Epidemiology, School of Public Health, Shandong University & Shandong University Center for Suicide Prevention Research, Jinan, Shandong China; 2 Department of Preventive Medicine, School of Basic Medical Sciences, Shandong University of Traditional Chinese Medicine, Changqing, Jinan, Shandong China; 3 Department of Social Psychology, Faculty of Human Ecology, Putra University of Malaysia, Serdang, Selangor Malaysia; 4 Department of Psychology, One UTSA Circle, The University of Texas at San Antonio, San Antonio, TX USA

**Keywords:** Reliability, Validity, Future disposition Inventory-24, Hopelessness, Suicide

## Abstract

**Background:**

This study was designed to assess the factor structure, internal consistency reliability, and preliminary psychometric properties of the Chinese version of the Future Disposition Inventory-24 (FDI-24) in a large sample of Chinese university students.

**Methods:**

We translated the English version of the Future Disposition Inventory-24 (FDI-24) into Chinese and examined its factor structure, estimates of internal consistency reliability, and psychometric properties in a representative sample of university students. In particular, students (*N* = 2,074) from two universities in Shandong Province in China were identified using the multi-stage stratified sampling method. In addition to the FDI-24, we collected preliminary data using self-report instruments that included the Beck Hopelessness Scale (BHS) and a general sociodemographic information questionnaire.

**Results:**

The results of the internal consistency reliability estimates were adequate regarding the scores on the three FDI-24 subscales: Cronbach’s alpha = .89–.97, Omega total = .85–.96, Revelle’s Omega total = .88–.96, the greatest lower bound (GLB) = .89–.96 and Coefficient *H* = .86–.94. Bivariate correlation analyses showed evidence for criterion and discriminant validity. The 3-factor oblique-Geomin-rotation solution accounted for 62.92% of the total variance in the exploratory factor analysis (EFA). The exploratory structural equation modeling (ESEM) result showed that the 3-factor model provided adequate fit statistics for the sample data: the robust comparative fit index (R-CFI) was .959, robust Tucker Lewis index (R-TLI) was .946 and robust root mean square error of approximation (R-RMSEA) was .090.

**Conclusion:**

The FDI-24 has a satisfactory factor structure, reliability estimates, and satisfactory evidence of concurrent validity estimates for students with different demographic and cultural backgrounds. The FDI-24 holds promise for use in future investigations with Chinese students.

## Background

Suicide remains an important public mental health concern for Western and non-Western nations. In particular, as reported by the World Health Organization (WHO) in 2014 [1], among young people aged 15–29 years, suicide remains the second leading cause of death globally. In China, in particular, suicide-related behaviors tend to be acute and serious mental health problems among young adults [1]. To date, a majority of the studies in the existing suicide literature tend to focus on psychopathology or risk factors, such as mood disorders [2, 3], anxiety disorders [4], and specific constructs, including hopelessness [5, 6], anger [7, 8], and loneliness [9, 10].

Hopelessness has been identified as a critical risk factor in the assessment of suicidal intentions and behaviors among students [11]. In recent years, several self-report instruments have been designed for the assessment of risk factors that are associated with suicide-related behaviors. As an example, according to Beck’s hopelessness theory of suicide, hopelessness is generally conceptualized as a pessimistic attitude or expectation about future life events; that is, it is generally considered one of the core cognitive vulnerability factors for suicide [12]. Indeed, previous studies have shown that prolonged and severe feelings of hopelessness may lead to high incidents of suicide-related behaviors [13]. Additionally, hopelessness has been identified as a moderator or mediator of the association between various psychological symptoms and suicide-related behaviors, even extending significantly beyond depression in predicting the severity of suicidal intent [14–16]. Furthermore, findings from theories such as strain theory of suicide [17] have demonstrated that individuals experiencing intolerable pain, hopelessness, and psychological strain might engage in high-risk suicide-related behaviors (e.g., suicide attempts). Therefore, it is of great significance to continue to evaluate the role of the hopelessness construct in assessing suicide-related behaviors.

In the past few years, self-report instruments have been developed and validated for the purpose of screening or assessing the hopelessness construct. As a notable example, the Beck Hopelessness Scale (BHS), developed 40 years ago, has been widely used to measure feelings of hopelessness in clinical and nonclinical samples [18–21]. Recently, however, Gutierrez and Osman [22] have argued for the inclusion of both risk and protective factors simultaneously in the assessment of suicide-related behaviors. Although some items regarding future prospects and pessimistic statements are included in the BHS, only a single dimension with a mix of positively worded (e.g., “I look forward to the future with hope and enthusiasm”) and negatively worded (e.g., “My future seems dark”) items underlies this given scale. We assume that the multiple aspects of positive and negative thinking may be differentially associated with hopelessness in individuals [23, 24]. To derive a total scale score for the BHS, nine of the positively worded items are reverse-scored (see comments by [25, 26]). It has also been noted that the BHS lacks content specificity for the assessment of suicide-related behaviors [27].

One self-report instrument receiving increasing attention in the extant literature in the assessment of future-related events is the Future Disposition Inventory–24 (FDI-24) [28]. In particular, the FDI-24 is designed to address some of the substantive psychometric limitations of existing self-report instruments, such as the BHS. In brief, the FDI-24 conceptualizes future events in terms of a *future disposition* along three correlated domains. The Positive domain of the FDI-24 focuses on responses such as optimism, plans, satisfaction with the future and determination in handling difficulties. The Negative domain focuses on feelings of worry, cognitive rigidity and life dissatisfaction, and the Suicide Orientation domain comprises suicidal rumination, ideation and the wish to die. Each item is scored on a 5-point Likert-type scale, ranging from 1 (*not at all true of me*) to 5 (*extremely true of me*). None of the 24 items is reverse-scored. Successfully used in some Western studies, the factor structure of the FDI-24 has been established, and its strong psychometric properties in adult and adolescent samples have been accepted [29–31]. However, it is unknown whether this instrument can be used to evaluate the construct of *future disposition* among university students in China. Thus, the specific aims of the current study were as follows. First, we examined the replicability of the 3-factor oblique solution of the FDI-24 in a large convenience sample of nonclinical participants. Second, we confirmed the structure of the final solution in the second half of the sample (referred to as *cross-validation* for purposes of the analyses). Third, we evaluated evidence of internal consistency reliability for scores on each domain. Fourth, we assessed the differential correlates of the FDI-24 domains.

## Methods

### Sample and procedure

Using a multi-stage stratified sampling procedure, the study participants consisted of a sample of undergraduate students recruited from two convenient large public medical universities in Jinan City, Shandong Province, eastern China. We selected two similar faculty colleges as the primary sampling unit from each university. Stratified on the basis of grade, three or four classes from each grade were randomly selected to be the secondary sampling units. Grade selection ranged from freshman to senior, with consideration of students’ absence for hospital internships. All students in the sampled classes present on the day of the survey were invited to complete the questionnaires. Professionals trained in instrument use and validation supervised the in-class survey administration (i.e., paper-pencil). In addition to the demographic questionnaire, the participants completed the Chinese versions of the Beck Hopelessness Scale [32] and the FDI-24. Of the 2,197 self-report questionnaires that were handed out, 2,074 were completed with no missing items on any of the study instruments. The sample, including 1,368 female students and 706 male students, had a mean age of 19.79 ± 1.39 years (female, mean age = 19.75 ± 1.33; male, mean age = 19.86 ± 1.50). The sample consisted of 574 (27.7%) freshmen, 521 (25.1%) sophomores, 619 (29.8%) juniors, and 369 (17.4%) seniors. In terms of ethnic composition, most of the participants were Han Chinese (1939, 93.5%), and 135 (6.5%) were from minority races. Before attending college, 1015 (48.9%) of them lived in an urban area, and 1059 (51.1%) lived in a rural environment. Preliminary analyses showed no statistically significant differences between separate units on demographic variables, such as gender, age, nationality, academic level and residency, all *p* values > .05.

### Future disposition inventory (FDI-24): Chinese version

Because this instrument has not been used previously with Chinese samples, the initial goal was to construct a Chinese version. Accordingly, we invited two bilingual experts specialized in mental health to guide the translation process of the FDI-24 instructions and items. To ensure appropriate and equivalent meanings and clarity of expressions, one expert translated all 24 items and instructions from English into Chinese, and the other expert translated the Chinese items and instructions back into English without being provided with the original instrument [33, 34]. After several rounds of discussion and revision, the final Chinese version of the instrument (i.e., retaining all 24 items) was adopted for use in the current study.

### The Beck hopelessness scale (BHS)

The BHS is a 20-item self-report instrument that is designed to assess negative attitudes about future events. Thus, it is considered a theoretically valid instrument for evaluating criterion validity estimates of scores on the FDI-24. Due to its development and good construct validation, this instrument has been widely used and translated into various languages. The instrument has strong estimates of test-retest reliability and construct validity in Chinese samples [32]. In brief, the BHS includes nine positively worded items and 11 negatively worded items concerning negative attitudes about the future. The total score of 20 items (each of which ranges from 1 to 5 points, with reverse scoring) is usually derived to evaluate levels of the hopelessness construct; higher total scores represent extreme levels of hopelessness. Used as a criterion-related validation instrument in the current study, the estimate of internal consistency of the BHS score for the study sample was adequate (Cronbach’s alpha = .90; average inter-item correlation [AIC] = .314).

### Statistical methods

The Cronbach’s alpha, Omega coefficients, Coefficient *H* and the greatest lower bound (GLB) procedures were used with the sample data to evaluate the estimate of internal consistency for scores on the self-report instruments.

Pearson correlations between scores on the BHS and the three domains of the FDI-24 were computed to examine the evidence for criterion-related validity. Based on the FDI-24 score distribution, a *t*-test was used to compare differences in the mean scores between extreme groups (the groups with the top 27% of scores and the bottom 27% of scores, respectively) [35, 36], and the method of Cohen’s *d* was applied to compute the discriminating power of subscales and to distinguish ability at different levels. These methods, used in a general student population, were able to distinguish extreme values to help define the high-scoring group members with a disposition of future thinking.

Participants were randomly divided into two groups by statistical software to evaluate further evidence for construct validity. Specifically, Exploratory Factor Analysis (EFA) was conducted with data obtained from one group, and Confirmatory Factor Analysis (CFA) was used with data from the other group. Finally, we retested the whole sample in fitting a confirmatory 3-factor model by exploratory structural equation modeling (ESEM; implemented in the Mplus 7.4 statistical software). The coefficient calculation was available in R packages. Considering the non-normal distribution of the scale item scores, we adopted the method of maximum likelihood means-adjusted estimator (Robust) to conduct the analyses. The *х*^*2*^*/df v*alue, robust comparative fit index (R-CFI), robust Tucker Lewis index (R-TLI), robust root-mean-square error of approximation (R-RMSEA), and its 95% *CI* were used to assess fit estimates for the 1-factor, 2-factor, and 3-factor solutions [37].

All statistical significance levels were set at a *p* value of .05.

### Ethics approval and consent to participate

The study was overseen by and obtained signed ethics approval from the institutional review board of the Ethics Committee at the School of Public Health, Shandong University (No. 20161103). All study participants gave voluntary verbal consent to participate in the anonymous survey after receiving an explanation of the study design and reading the questionnaire instructions prior to responding.

## Results

### Reliability

The Cronbach’s alphas for each scale score of the FDI-24 were high (.89–.97), indicating good internal consistency reliability (see Table 1) [38]. Measures of reliability alongside Cronbach’s alpha, Omega coefficients, the greatest lower bound (GLB) and Coefficient *H* were reported on the three subscales, performing well in assessing the reliability of scales. The corrected item-total correlations beyond .30 are acceptable based on an empirical study [39], as shown in Table 2, although the correlations for each scale were higher than what we expected. Specifically, the range was .741 to .828 for the Positive Focus scale items, .737 to .884 for the Suicide Orientation scale items, and .629 to .853 for the Negative Focus scale items, respectively. Taken together, all correlation coefficient values were statistically significant (*p* < .001).

**Table 1:** Internal consistency of dimension-total scores of the future disposition inventory-24

Dimension	Items	Mean ± *SD*	*α*	*ω*	Revelle’s *ω*	GLB	*H*
Positive focus	8	30.96±7.26	0.94	0.92	0.94	0.94	0.92
Suicide orientation	8	12.17±7.00	0.97	0.96	0.96	0.96	0.94
Negative focus	8	16.48±6.30	0.89	0.85	0.88	0.89	0.86

*α*: Cronbach’s alpha; *ω*: Omega total; Revelle’s *ω* = Revelle’s Omega total; GLB: the Greatest Lower Bound; *H*: Coefficient *H*

**Table 2:** Internal consistency of item-dimension scores of the future disposition inventory-24

Abbreviated content items	Mean ± *SD*	*r*
Positive focus		
1. I expect things to turn out better.	4.08±1.14	.809
2. I plan to work harder to make things better.	4.03±1.11	.828
5. I plan to deal better with most of the setbacks.	3.61±1.19	.747
7. I expect to enjoy the results or outcomes of all my hard work.	3.90±1.17	.794
10. I remain determined to deal better with demands on me.	3.84±1.13	.823
11. I expect to be happier and more content with my life.	3.99±1.12	.822
13. I plan to look at the positive side of my life.	3.74±1.12	.783
15 I intend to succeed in working through most personal problems.	3.77±1.16	.741
Suicide orientation		
3. I think that by ending my life, all problems will go away.	1.63±1.14	.737
6. I think life is not worth living.	1.58±1.08	.830
8. I think things will get better if I were dead.	1.51±1.03	.865
9. I think people would be better off without me.	1.57±1.12	.798
17. I sometimes wish I were dead.	1.52±1.06	.873
18. I think I would be better off dead.	1.45±1.03	.884
22. I wish I could succeed at attempts to kill myself.	1.42±0.98	.816
23. I have nothing to lose by ending my life.	1.48±1.05	.808
Negative focus		
4. I worry that things will never go well.	1.76±1.10	.665
12. I get confused and uncertain.	2.49±1.15	.853
14. I doubt whether things will ever get better.	2.39±1.14	.697
16. I have a hard time imagining that things will ever get better for me.	1.80±1.13	.691
19. I wonder whether I would ever be satisfied.	2.34±1.20	.629
20. I fear that I will run into more difficulties.	2.16±1.14	.762
21. All I can see ahead of me are hardships.	1.68±1.03	.730
24. I often doubt whether I will ever have control.	1.86±1.15	.765

### Criterion validity

The Beck Hopelessness Scale (BHS) total scale score was used as the criterion measure in our analyses. It might weaken the effects of opposite directions to evaluate global future attitudes, thoughts and feelings using just a single orientation of an inventory. Thus, for a better understanding of the correlations between scores on the BHS total scale and the FDI-24 subscales, we conducted separate correlation analyses with the specific FDI-24 scales’ scores. Consequently, we found that the Positive Focus scale score was negatively and significantly associated with the BHS total scale score (*r* = −.53, *p* < .001). The Negative Focus scale score was positively and significantly associated with the BHS total scale score (*r* = .49, *p* < .001). The Suicide Orientation scale score was also positively and significantly associated with the BHS total scale score (*r* = .40, *p* < .001), suggesting evidence for adequate criterion validity of scores on the FDI-24 scales.

### Discrimination

An independent samples *t*-test was used to compare the different means between the high- and low-scoring groups. For the Positive Focus scale of FDI-24 inventory data, we observed that the mean of the group with the top 27% of scores (27.41 ± 6.29) was significantly lower than that of the group with the bottom 27% of scores (30.57 ± 8.10, *p* < .001). As expected, similar results were obtained when the analyses were undertaken with the other two specific FDI-24 subscales scores (See Table 3). A percentage of 27% was used because this value was able to maximize differences in normal distributions while providing enough cases for analysis. Cohen’s *d* as a measure of group differences was also reported in the result, displaying acceptable discriminating power of each scale.

**Table 3:** Discriminant validity of the future disposition inventory-24

Dimension	High 27% Mean ± *SD*	Low 27% Mean ± *SD*	*t-value*	*p-value*	*Cohen’s d*
Positive focus	27.41 ± 6.29	30.57 ± 8.10	-7.677	<.001	-.436
Suicide orientation	9.18 ± 2.51	19.11 ± 9.28	-26.337	<.001	-1.461
Negative focus	12.47 ±3.22	23.65 ± 5.44	-44.453	<.001	-2.501

### Construct validity

The items in the Chinese version of the FDI-24 were first analyzed using the oblique-Geomin-rotation procedure. The results of Bartlett’s Test of Sphericity and the Kaiser-Meyer-Olkin (KMO) test (*х*^*2*^ = 17260, *p* < .001 and the KMO = .942) showed that the present sample was well suited for factor analysis. Based on the results of the screen plot and the criterion of eigenvalues greater than 1, the three-factor solution was retained, accounting for 62.92% of the total variance.

We used the recommended cutoff score of .50 or higher to guide detailed interpretations of the item-factor loadings [40]. In the scale designer’s initial analysis, the pattern of loadings on each domain was eight items [28]. However, using a cutoff score of .50 or higher, we found that, in EFA, Item 4 (“I worry that things will never go well for me no matter what I do”) from the Negative Focus factor had a high loading on the Suicide Orientation factor, at .624. Furthermore, Item 16 (“I have a hard time imagining that things will ever get better for me in the future”) had a loading that was lower than expected on the Negative Focus factor. For the Suicide Orientation factor, we found that Item 22 (“I wish I could succeed at attempts to kill myself”) and Item 23 (“I feel I have nothing to lose by ending my life”) had higher loadings on the Negative Focus factor than on the expected Suicide Orientation factor (see Table 4).

**Table 4:** Factor loadings from the exploratory and confirmatory factor analyses

	EFA(n_1_=1037)			CFA(n_2_=1037)		
	1	2	3	1	2	3
	Factor1			Factor1		
1	0.733	-0.443	0.237	0.928		
2	0.754	-0.393	0.186	0.934		
5	0.741	0.014	-0.079	0.740		
7	0.771	-0.089	-0.029	0.799		
10	0.846	0.060	-0.145	0.867		
11	0.833	-0.004	-0.135	0.841		
13	0.767	0.017	-0.167	0.800		
15	0.750	0.009	-0.090	0.714		
		Factor2			Factor2	
3	-0.030	0.737	0.114		0.789	
6	-0.090	0.743	0.196		0.892	
8	-0.124	0.828	0.122		0.915	
9	-0.101	0.754	0.130		0.867	
17	-0.079	0.735	0.295		0.936	
18	-0.101	0.743	0.290		0.969	
22	-0.248	0.445	0.523		0.937	
23	-0.220	0.451	0.525		0.897	
			Factor3			Factor3
4	-0.057	0.624	0.213			0.841
12	0.079	0.158	0.501			0.561
14	0.082	0.178	0.569			0.625
16	-0.106	0.473	0.395			0.825
19	0.112	-0.132	0.781			0.508
20	-0.028	0.022	0.809			0.680
21	-0.121	0.281	0.623			0.848
24	-0.102	0.244	0.633			0.808

EFA = Exploratory Factor Analysis; CFA = Confirmatory Factor Analysis; n1 and n2 were two random sample

We conducted confirmatory factor analysis (CFA) to confirm the fit of the three-factor model with the second sample data. Table 5 shows fit estimates for the different structural models, providing support for the construct validity of the correlated three-factor model. Regarding the CFA factor loadings, each factor was composed of eight items, similar to the original solution. The range of the standardized factor loadings for each factor is presented: .714 to .934 for Positive Focus, .789 to .969 for Suicide Orientation, and .508 to .848 for Negative Focus (see Table 4).

**Table 5:** Fit estimates for the different structural models in EFA, CFA and ESEM

	*х* ^*2*^	*df*	R-CFI	R-TLI	R-RMSEA	R-RMSEA 95% CI
Exploratory Factor Analysis (EFA)						
1-factor	6824.335^a^	252	0.852	0.837	0.159	(0.155-0.162)
2-factor	2845.755 ^a^	229	0.941	0.929	0.105	(0.102-0.108)
3-factor	1848.354 ^a^	207	0.963	0.951	0.087	(0.084-0.091)
Confirmatory Factor Analysis (CFA)						
3-factor	2618.928 ^a^	249	0.945	0.939	0.096	(0.093-0.099)
Exploratory Structural Equation Modeling (ESEM)						
1-factor	13558.135 ^a^	252	0.846	0.832	0.160	(0.157-0.162)
2-factor	5775.137 ^a^	229	0.936	0.923	0.108	(0.106-0.110)
3-factor	3713.35 ^a^	207	0.959	0.946	0.090	(0.088-0.093)

*х*^*2*^ Chi-square test value, *df* degree of freedom, *R-CFI* robust comparative fit index, *R-TLI* robust Tucker Lewis index, *R-RMSEA* robust root-mean-square error of approximation, *CI* confidence interval, ^a^*p*<.05

The ESEM fit indices of the 3-factor model also indicated adequate fit of the model to the sample data in Table 5. As shown in Table 6, most of the items had adequate loadings on the proposed original factors [28], while Items 4, 16 and 23 had higher loadings on factors other than the original.

**Table 6:** Factor loadings from the Exploratory Structural Equation Modeling (ESEM)

		ESEM(n=2074)	
	1	2	3
	Factor1		
1	0.731	-0.462	0.272
2	0.742	-0.427	0.223
5	0.742	0.028	-0.108
7	0.774	-0.074	-0.036
10	0.851	0.024	-0.121
11	0.833	-0.004	-0.118
13	0.776	0.027	-0.173
15	0.754	0.032	-0.081
		Factor2	
3	-0.002	0.733	0.145
6	-0.109	0.742	0.183
8	-0.081	0.826	0.143
9	-0.093	0.746	0.153
17	-0.095	0.745	0.266
18	-0.143	0.737	0.273
22	-0.293	0.425	0.517
23	-0.228	0.449	0.497
			Factor3
4	-0.046	0.602	0.255
12	0.098	0.177	0.511
14	0.087	0.218	0.539
16	-0.095	0.494	0.363
19	0.098	-0.122	0.765
20	-0.003	0.008	0.817
21	-0.130	0.289	0.598
24	-0.080	0.245	0.638

As expected, the correlation between the Positive Focus factor and the Suicide Orientation factor was negative and high at −.569. The correlation between the Suicide Orientation factor and the Negative Focus factor was positive and high at .873. The correlation between the Positive Focus factor and the Negative Focus factor was negative and moderate at −.372.

## Discussion

In this study, the main findings are as follows. First, the Future Disposition Inventory-24 (FDI-24) domains had satisfactory internal consistency reliability estimates in a sample of Chinese students with different cultural backgrounds. Second, we found, using scores on the Beck Hopelessness Scale (BHS), that the FDI-24 scale scores had acceptable criterion validity. Third, the estimate of discrimination, assessed by examining scores between high- and low-scoring groups, could be considered adequate. Fourth, evaluation of the three-factor model with conventional goodness-of-fit statistics showed replicability of the three-factor solution of the items in the study samples, providing support for construct validity.

Although we found that the FDI-24 evaluated the same three domains of the future disposition construct for the Chinese-speaking students, minor differences were observed between these groups on four of the FDI-24 items. For the Chinese-speaking students, factor loadings of two future suicide orientation items (i.e., Items 22 and 23) were high on the Negative Focus scale for the future (e.g., worry about the future). It should also be noted that two of the Negative Focus items (Items 4 and 16) had loadings of .40 or higher on the Suicide Orientation scale in the EFA and ESEM (see Tables 4 and 6). However, when the 3-factor oblique model was examined in the validation sample, we found that all four of these items (Items 4, 16, 22, and 23) had high, positive and significant loadings. Accordingly, we did not exclude any of the FDI-24 items in the analyses. Future measurement invariance investigations with independent American and Chinese samples might identify specific items that are considered as country specific.

The present study is the first to examine the psychometric properties of the FDI-24 in a sample of Chinese university students. As with the American student samples, we found that most of the item-total correlations for three domains of the FDI-24 were greater than .70. In particular, in a study by Osman and colleagues [28], the reliability estimates for the three subscales’ scores were moderate to high (i.e., ranging from .86 to .93). Ballard et al. [31] also reported good internal consistency reliability estimates of .86 and .89 for two domains of the FDI-24 among undergraduate psychology students. Likewise, high reliability estimates of the scale scores were reported for military personnel samples in the United States [41]. An additional strength of the current study relates to the use of data from a large sample of study participants. In addition, systematic steps that involved the use of both exploratory and confirmatory procedures were undertaken to address the specific goals of the study.

Despite the strengths noted, several limitations were also prominent. First, the findings need to be replicated in other Western and non-Western clinical and nonclinical samples. Second, all the study participants were college-age students who presented with low to moderate risk factors for suicide-related behaviors. It might have been useful to screen study participants for suicide-related behaviors, including the frequency of suicide ideation, history of suicide threats, and other forms of psychopathology. It is worth noting, however, that the sample was composed of medical students, who tend to present with higher prevalence rates of depression and anxiety symptoms as well as higher levels of psychological distress than in the general population [42]. Despite these limitations, this is the first study to demonstrate acceptable internal consistency reliability and validity estimates of FDI-24 scores in Chinese university students. These findings support the adequacy of the psychometric properties of the FDI-24 scale scores in Chinese culture.

## Conclusion

The reliability and validity of the FDI-24 were supported by the data from a large Chinese university student sample. More studies with nationwide samples are needed to replicate current findings and to further examine other psychometric properties of the FDI-24.

## Abbreviations

AIC: (average inter-item correlation)
BHS: Beck Hopelessness Scale
CFA: Confirmatory Factor Analysis
EFA: Exploratory Factor Analysis
ESEM: Exploratory Structural Equation Modeling
FDI-24: Future Disposition Inventory-24
KMO: Kaiser-Meyer-Olkin
NF: Negative Focus
PF: Positive Focus
R-CFI: Robust comparative fit index
R-RMSEA: Robust root-mean-square error of approximation
R-TLI: Robust Tucker Lewis index
SO: Suicide Orientation
